# 

**Published:** 1911-09

**Authors:** 


					rn
SEPTEMBER, 1911.
Vol. XXIX.  No. 11R
the/1 i"bTTa K Vj
BRISTOL 131i ' I
SURGEOS-GciuRALS l/r r XE j
MEDICO-CH1W^RtrtCAr
TOU
' PUBLISHED V
RNAL
PUBLISHED UNDER THE AUSPICES OF
THE BRISTOL MEDICO-CHIRURGICAL SOCIETT.
Editor: R. SHINGLETON SMITH, M.D., B.Sc.
WITH WHOM ARE ASSOCIATED
J. MICHELL CLARKE, M.A., M.D.,
J. LACY FIRTH, M.S., M.D., JAMES SWAIN. M.S., M.D.
P. WATSON WILLIAMS, M.D., Assistant Editor.
Editorial Secretary: J. A. NIXON, B.A., M.B.
BRISTOL: J. W. ARROWSMITH LTD.
London: J. & A. Churchill, 7 Great Marlborough Street.
Price One Shilling and Sixpence.

				

